# Exploring the influencing factors of unmet palliative care needs in Chinese patients with end-stage renal disease undergoing maintenance hemodialysis: a cross-sectional study

**DOI:** 10.1186/s12904-023-01237-x

**Published:** 2023-08-05

**Authors:** Xuefei Wang, Yongzhen Mo, Yingying Yuan, Yi Zhou, Yan Chen, Juan Sheng, Jing Liu

**Affiliations:** 1 Jiangsu Province Official Hospital, Nanjing, Jiangsu China; 2 School of Nursing, Department of Medicine, Soochow University, Suzhou, Jiangsu China; 3 Nanjing BenQ Medical Center, Nanjing, Jiangsu China

**Keywords:** End-stage renal disease, Hemodialysis, Palliative care, Risk factors, Unmet need

## Abstract

**Background:**

The role of palliative care for end-stage renal disease (ESRD) patients have been proven in some developed countries, but it is still unclear in the mainland of China. In fact, patients with ESRD experience many unmet palliative care needs, such as physical, psychological, social and spiritual needs, but the factors influencing these needs have not investigated.

**Methods:**

A cross-sectional study was conducted at two hemodialysis centers in the mainland of China from January to September 2022. Convenience sampling was used to collect data on the participants' socio-demographics, clinical characteristics, the Palliative Care Outcome Scale (POS), the Dialysis Symptom Index (DSI), the Karnofsky Performance Status Scale (KPS), the Patient Health Questionnaire-9 item (PHQ-9), and the Social Support Rate Scale (SSRS). Data were analyzed using latent profile analysis, Kruskal–Wallis test, one-way analysis of variance (ANOVA), the chi-square test and multinomial logistic regression analysis.

**Results:**

Three hundred five participants were included in this study, and divided palliative care needs into three categories: Class 1, mild palliative care needs (*n* = 154, 50.5*%*); Class 2, moderate palliative care needs (*n* = 89, 29.2*%*); Class 3, severe palliative care needs (*n* = 62, 20.3*%*). Based on the analysis of three profiles, the influencing factors of unmet needs were further analyzed. Compared with Class 3, senior high school education, the household per capita monthly income < 2,000, low KPS scores, high PHQ-9 scores, and low SSRS scores were less likely to be in Class 1 (*OR* = 0.03, *P* = 0.012; *OR* = 0.003, *P* < 0.001; *OR* = 1.15, *P* < 0.001; *OR* = 0.55, *P* < 0.001; *OR* = 1.35, *P* = 0.002; respectively) and Class 2 (*OR* = 0.03, *P* = 0.007; *OR* = 0.05, *P* = 0.011; *OR* = 1.10, *P* = 0.001; *OR* = 0.60, *P* = 0.001; *OR* = 1.32, *P* = 0.003; respectively), and high symptom severity were less likely to be in Class 1 (*OR* = 0.82, *P* = 0.001). Moreover, compared with Class 1, the household per capita monthly income < 2,000 (*OR* = 16.41, *P* < 0.001), high symptom severity scores (*OR* = 1.12, *P* = 0.002) and low KPS scores (*OR* = 0.95, *P* = 0.002) were more likely to be in Class 2.

**Conclusions:**

This study showed that almost half of ESRD patients receiving MHD presented moderate to severe palliative care needs, and the unmet needs were mainly affected by education level, financial pressure, functional status, symptom burden and social support. In the future, it is important to identify the populations with the greatest need for palliative care and consider the influencing factors of unmet needs from a comprehensive perspective, so as to help them improve health-related quality of life.

**Supplementary Information:**

The online version contains supplementary material available at 10.1186/s12904-023-01237-x.

## Background

Hemodialysis (HD) has become the mainstay of treatment for patients with end-stage renal disease (ESRD) [1]. By 2020, there were about 632,000 maintenance hemodialysis (MHD) patients in the mainland of China [2]. Although treatment can prolong survival, the current dominant healthcare delivery model for ESRD patients focus almost exclusively on optimizing the delivery of dialysis care, to the extent that patient needs other than dialysis treatment are largely ignored [3]. Notably, the long-term nature and complexity of the disease has a great impact on patients' physical, psychological, mental and daily life [4–6]. What’s more, previous studies showed that patients with ESRD near the end-of-life experienced increased rates of hospitalization and invasive treatment [7], as well as lower family ratings of the quality of end-of-life care [8].

Palliative care is a new nursing model aimed at alleviating patients' suffering and improving health-related quality of life (HRQOL) [9]. The positive effects of palliative care on symptom management [10], hospitalization rates [11], medical costs [11], and end-of-life care quality [12] in ESRD patients had been validated in some studies. Despite the concept of non-cancer palliative care has gradually attracted attention, the main object of palliative care in clinical practice is still advanced cancer patients in the mainland of China [13].

In particular, needs assessment is the first step in implementing palliative care [9]. Previous original studies on the palliative care needs of ESRD patients mainly focused on the content assessment of needs, which showed that the unmet palliative needs of ESRD patients were mainly reflected in physical, emotional, psychosocial, spiritual, informational and practical issues [14, 15]. However, it is not clear what factors influence the unmet need of patients.

A systematic review of palliative care needs for severe illnesses in Africa showed that individuals (patients and families), health and disease, environment, and treatment predicted patients' unmet needs [16]. In addition, the social ecological model with the holistic view as the core indicated that patients' unmet needs were influenced by the interactions among personal (e.g., demographics, physiological condition, psychological disposition), interpersonal (e.g., social support), community (e.g., proximity to suitable services & resources) and policy (e.g., available assistance for medical costs) [17]. On top of that, the model was often used to analyze the factors that contribute to the unmet needs of patients with advanced cancer and cardiovascular disease in an attempt to provide them with palliative and supportive care [17–19].

Although previous studies explored the influencing factors of palliative care needs for patients with other chronic malignant and non-malignant diseases (e.g., advanced cancer, Parkinson's disease), they analyzed the palliative care need score as a continuous variable, ignoring the heterogeneity of individual needs [20, 21]. Potential profile analysis is an individual-centered classification of samples based on different characteristics of individuals, and can show the proportion of people in each category [22]. Its classification accuracy is higher than the traditional classification method, and it has become a powerful tool to solve the problem of continuous variable classification, and has been widely used in the field of medicine and psychology [23–25].

In order to accurately identify the main subjects of palliative care and meet their physical, psychological, social and spiritual needs. The purpose of this study was to classify the level of palliative care needs of patients with ESRD undergoing MHD based on latent profile analysis, and to explore the influencing factors of unmet needs.

## Methods

### Study setting and participants

From January to September 2022, a cross-sectional study was conducted at the HD centers of BenQ Medical Center and Jiangsu Province Geriatric Hospital in Nanjing, Jiangsu Province, China. In general, MHD patients came to the hospital 2–3 times a week for HD treatment, and we recruited them when they came to the hospital.

After obtaining participants' consent, two trained personnel explained the purpose and content of the questionnaire to the participants. These questionnaires were mainly self-reported by the patients. When the patients could not complete these questionnaires independently (such as visual impairment), the researcher helped them complete these questionnaires. Ultimately, we recruited 305 MHD patients, which was sufficient to test the logistic regression analysis because the ideal sample size was at least 5–10 times each variable [26].

Eligible participants should meet the following criteria: 1) CKD stage V, with an estimated glomerular filtration rate (eGFR) ≤ 15 ml/min/1.73m^2^ (using Cockcroft-Gault Formula); 2) Duration of HD treatment ≥ 3 months, regular HD ≥ 2 times a week; 3) Age ≥ 18 years old; 4) Normal cognitive function and language communication. Participants will be excluded if suffering from other serious diseases such as cancer or recent traumatic events.

### Variables and measures

#### Socio-demographics and clinical characteristics

A self-designed questionnaire was used to collect general information on socio-demographics and clinical characteristics, including age, gender, education level, place of residence, marital status, per capita monthly household income, health insurance status, primary cause, duration of MHD since diagnosis, and some laboratory indicators (e.g., Serum phosphorus, Serum calcium, Hemoglobin, Intact parathyroid hormone and Clearance index of urea) for the last three months were sought from medical records.

#### The Palliative Care Outcome Scale (POS)

The Palliative Care Outcome Scale (POS) was widely used to investigate the palliative care needs of patients with chronic or progressive disease, regardless of their diagnostic and clinical setting [27–29]. The 10 items of the tool cover the domains related to palliative care, such as physical, emotional, mental, spiritual, information provision and support. Each item is scored from 0 (best) to 4 (worst), with a total score of 0–40 [30]. A higher score indicate the greater unmet needs [20]. The Chinese version of the POS had been proved to good reliability and validity (Cronbach’s α = 0.746) [31], and the Cronbach’s α in this study was 0.822. This scale is available for free from the POS website (https://pos-pal.org/maix/pos-downloads.php) [32].

#### The Dialysis Symptom Index (DSI)

The Dialysis Symptom Index (DSI) was commonly used to assess symptoms and severity in MHD patients [33]. The tool includes a total of 30 items that target specific physical or emotional symptoms. Enrolled participants were asked to report the presence (yes or no) of each symptom during the past week, and if the symptom was present, a 5-point Likert scale (1 = "not at all bothersome" to 5 = "bothers very much") was used to evaluate the severity of symptoms. Two scores were generated from the DSI [34, 35]. First, an overall symptom burden score was developed based on the total number of reported symptoms, with scores ranging from 0 to 30. Second, an overall symptom severity score was generated by summing the severity scores for each reported symptom, with scores ranging from 0 to 150. The Mandarin version of the DSI had shown good reliability and validity (Cronbach’s α = 0.87) [36]. The Cronbach’s α for the tool in this study was 0.844.

#### The Karnofsky Performance Status Scale (KPS)

The Karnofsky Performance Status Scale (KPS) was a validated tool for assessing functional status, and had been used extensively in dialysis patients [36, 37]. The patient's functional status was rated on a scale of 0 (“death”)-100 (“no evidence of disease, no symptoms”), and with higher scores indicating better health.

#### The Patient Health Questionnaire-9 item (PHQ-9)

The Patient Health Questionnaire-9 item (PHQ-9) was used to assess depressive symptoms experienced in the past two weeks [38]. The nine items of the tool were based on the criteria for evaluating symptoms of major depression in the Diagnostic and Statistical Manual of Mental Disorders (DSM-IV). Each item was scored on a 4-point Likert scale ranging from 0 (“not at all”) to 3 (“nearly every day”). The total range of 0–27, with higher scores indicating higher levels of depression. The tool had been verified in Chinese population (Cronbach's α = 0.842) [39]. In this study, the Cronbach's α of the PHQ-9 was 0.827.

#### The Social Support Rate Scale (SSRS)

The Social Support Rate Scale (SSRS) was designed by Xiao [40], and used to evaluate the level of social support. The scale included 3 dimensions (objective support, subjective support and utilization of social support) and 10 items. Questions 1–4, 8–10 were rated on a 4-point Likert scale, with 1 representing "cannot get support" and 4 representing "get sufficient support". Question 5 calculated the total score of the five items:A, B, C, D, E. Each item used a 4-point Likert scale, with 1 representing "no support" and 4 representing "full support". If the answers to questions 6 and 7 were "no source," participants scored 0; If the answers were "from the following sources," participants rated the number of listed sources. The total score ranged from 12 to 66, with higher scores indicating better levels of social support. The Cronbach's α of the SSRS in Chinese samples was 0.80 [41], and Cronbach's α for the scale in this study was 0.846.

### Data analyses

Mplus 8.3 software was used for the latent profile analysis (LPA) [42]. The model fit evaluation criteria were Akaike Information Criterion (AIC), Bayesian Information Criterion (BIC), sample-size-adjusted BIC (SABIC), Entropy, Lo-Mendell-Rubin (LMR) and Bootstrap Likelihood Ratio Test (BLRT) [43]. Smaller values of AIC, BIC, SABIC suggest an improved model fit. Entropy is a way of assessing the effect produced by each group of LPA, with a value equal to or greater than 0.80 indicating better results. LMR and BLRT were used to compare the fitting differences of the model, if the *P*-value of these two indicators reaches a significant level, it means that *k* profile solution is better than *k*-1 profile solution.

After determining the best latent profile model, statistical analyses were performed using the SPSS 26.0 software. Continuous variables with normal distribution were described by mean and standard deviation, non-normal distribution was described by median and interquartile range. Categorical variables were described by frequency and percentage. Univariate analysis was performed by Kruskal–Wallis test, one-way analysis of variance (ANOVA), or the chi-square test. Multinomial logistic regression analysis was used for multivariate analysis. *P* < 0.05 was considered statistically significant.

### Ethical consideration

The study was approved by the ethics committee of Jiangsu Province Official Hospital (2022–006). Participants were explained with the purpose, process, duration, possible benefits and risks of participation. All participants could withdraw from the study at any time. Oral or written consent was obtained from all participants prior to commencement of the study.

## Results

### Sample characteristics

Table 1 presented the socio-demographic and clinical characteristics of the sample. In brief, 163 participants (53.4*%*) were the older; 63*%* were male; 46.9*%* had junior high school and below; 92.1*%* lived in urban areas; More than four-fifths of the participants were married; Participants' primary cause mainly included hypertensive nephropathy (27.2*%*), glomerulonephritis (26.6*%*), and diabetic nephropathy (25.9*%*); Nearly half of the participants were on dialysis for 2–5 years.

**Table 1:** Socio-demographic and clinical characteristics of the sample (*N* = 305)

Variables	*n (%*) or *M* (*P*_25_, *P*_75_)
Age, years	67.0 (59.0, 72.0)
< 65	142 (46.6)
≥ 65	163 (53.4)
Gender	
Male	192 (63.0)
Female	113 (37.1)
Education level	
Junior high school and below	143 (46.9)
Senior high school	87 (28.5)
Senior high school above	75 (24.6)
Place of residence	
Rural	24 (7.9)
Urban	281 (92.1)
Marital status	
Married	252 (82.6)
Unmarried, divorced or widowed	53 (17.4)
Per capita monthly household income	
< 2,000 Yuan RMB (approximately 280.4341 US$)	91 (29.8)
2,000–5,000 Yuan RMB (approximately 280.4341–701.0853 US$)	107 (35.1)
> 5,000 Yuan RMB (approximately 701.0853 US$)	107 (35.1)
Health insurance	
No	6 (2.0)
Yes	299 (98.0)
Primary cause	
Glomerulonephritis	81 (26.6)
Diabetic nephropathy	79 (25.9)
Hypertensive nephropathy	83 (27.2)
Polycystic kidney/nephrotic syndrome	28 (9.2)
unknown	34 (11.2)
Duration of MHD since diagnosis, years	
< 2	63 (20.7)
2–5	151 (49.5)
5–10	53 (17.4)
> 10	38 (12.5)
Serum phosphorus (mmol/L)	1.76 (1.42, 2.18)
Serum calcium (mmol/L)	2.28 (2.16, 2.42)
Hemoglobin (g/L)	110.0 (97.5, 120.0)
Intact parathyroid hormone (pg/ml)	169.70 (87.44, 281.9)
Clearance index of urea (Kt/V)	1.47 (1.28, 1.65)

*MHD* Maintenance hemodialysis

### Latent profile analysis of the POS

The total mean POS score of MHD patients was 17.50 (SD = 7.54), and the score of each item was shown in Additional file: Table S1. The 10 items of the POS were taken as the explicit indicators, and 1–4 potential profile models were successively selected for exploratory potential profile analysis. The results (Table 2) showed that the AIC, BIC, and SABIC values decrease as the number of profiles increases from the 2-profile model to the 4-profile model, with Entropy > 0.8. However, the LMR value of the 4-profile model was not statistically significant (*P* > 0.05), indicating that the 3-profile model was superior to the 4-profile model. Hence, the 3-profile model was identified as the best potential profile model. Additionally, in order to verify the reliability of the classification results, the attribution probability of the three class samples in each class was calculated. Table 3 showed that the correct classification probabilities of the three classes were 97.7*%*, 92.9*%* and 94.1*%*, respectively, indicating a good discriminability and reliable classification of the 3-profile model.

**Table 2:** Fit indices of latent profile analysis of the POS (*N* = 305)

Model	AIC	BIC	SABIC	Entropy	LMR (*P)*	BLRT (*P)*
1-profile	9556.563	9630.969	9567.539	-	-	-
2-profile	8690.989	8806.319	8708.002	0.916	< 0.001	< 0.001
3-profile	8466.385	8622.638	8489.434	0.907	0.007	< 0.001
4-profile	7984.857	8182.033	8013.943	1.000	0.640	< 0.001

*POS* Palliative care outcome scale, *AIC* Akaike information criterion, *BIC* Bayesian information criterion, *SABIC* Sample-size adjusted BIC, *LMR* Lo-Mendell-Rubin, *BLRT* Bootstrap likelihood ratio test

**Table 3:** Three latent class attribution probabilities

Latent classes	Class 1	Class 2	Class 3
Class 1	0.977	0.023	0.000
Class 2	0.036	0.929	0.034
Class 3	0.000	0.059	0.941

**Fig. 1:**
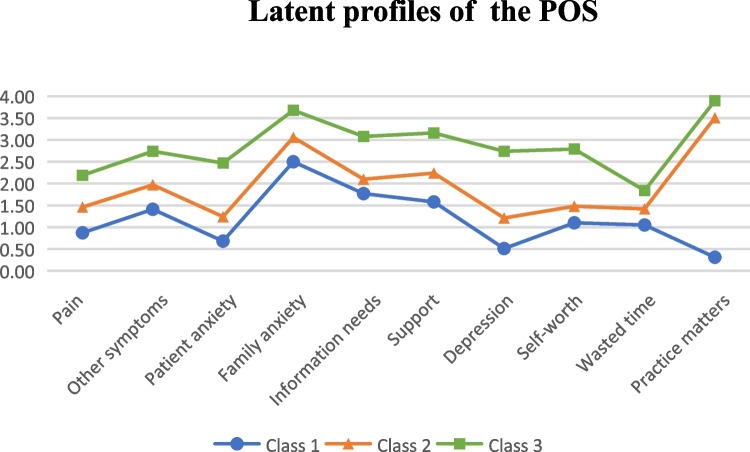
Latent profiles of the POS

Three classes of the POS were depicted in Fig. 1. Class 1 was named mild palliative care needs because it scored relatively low on each item of the POS (*n* = 154, 50.5*%*), with total mean POS scores of 11.77 (SD = 3.98). Similarly, Class 2 and Class 3 were named as moderate palliative care needs (*n* = 89, 29.2*%*) and severe palliative care needs (*n* = 62, 20.3*%*), with total mean POS scores of 19.67 (SD = 3.52) and 28.60 (SD = 3.30), respectively.

### Analysis of associated factors of the POS profiles

Table 4 presented the differences in the general information and major variables of the participants based on the POS profiles. Multinomial logistic regression analysis was conducted with statistically significant variables in univariate analysis as independent variables, and three classes of the POS as dependent variables. The results were shown in Table 5. Specifically, compared with Class 3, senior high school education, the household per capita monthly income < 2,000, low KPS scores, high PHQ-9 scores, and low SSRS scores were less likely to be in Class 1 (*OR* = 0.034, *P* = 0.012; *OR* = 0.003, *P* < 0.001; *OR* = 1.151, *P* < 0.001; *OR* = 0.553, *P* < 0.001; *OR* = 1.351, *P* = 0.002; respectively) and Class 2 (*OR* = 0.033, *P* = 0.007; *OR* = 0.045, *P* = 0.011; *OR* = 1.097, *P* = 0.001; *OR* = 0.603, *P* = 0.001; *OR* = 1.323, *P* = 0.003; respectively), and high symptom severity scores were less likely to be in Class 1 (*OR* = 0.817, *P* = 0.001). What’s more, compared with Class 1, the household per capita monthly income < 2,000 (*OR* = 16.410, *P* < 0.001), high symptom severity (*OR* = 1.123, *P* = 0.002) and low KPS scores (*OR* = 0.953, *P* = 0.002) were more likely to be in Class 2.

**Table 4:** Univariate analysis of the POS among three classes (*N* = 305)

Variables	Class 1 (*n* = 154)	Class 2 (*n* = 89)	Class 3 (*n* = 62)	*χ*^*2*^ */H/F*	*P*
Age, years, *n* (*%*)				15.770	< 0.001
< 65	87 (61.3)	38 (26.8)	17 (12.0)		
≥ 65	67 (41.1)	51 (31.3)	45 (27.6)		
Gender, *n* (*%*)				5.239	0.073
Male	106 (55.2)	53 (27.6)	33 (17.2)		
Female	48 (42.5)	36 (31.9)	29 (25.7)		
Education level, *n* (*%*)				14.020	0.007
Junior high school and below	58 (40.6)	46 (32.2)	39 (27.3)		
Senior high school	48 (55.2)	24 (27.6)	15 (17.2)		
Senior high school above	48 (64.0)	19 (25.3)	8 (10.7)		
Place of residence, *n* (*%*)				15.188	0.001
Rural	3 (12.5)	13 (54.2)	8 (33.3)		
Urban	151 (53.7)	76 (27.0)	54 (19.2)		
Marital status, *n* (*%*)				2.379	0.304
Married	132 (52.4)	72 (28.6)	48 (19.0)		
Unmarried, divorced or widowed	22 (41.5)	17 (32.1)	14 (26.4)		
Per capita monthly household income, *n* (*%*)				87.850	< 0.001
< 2,000 Yuan RMB (approximately 280.4341 US$)	12 (13.2)	39 (42.9)	40 (44.0)		
2,000–5,000 Yuan RMB (approximately 280.4341–701.0853 US$)	61 (57.0)	33 (30.8)	13 (12.1)		
> 5,000 Yuan RMB (approximately 701.0853 US$)	81 (75.7)	17 (15.9)	9 (8.4)		
Health insurance					
No	1 (17.0)	4 (66.7)	1 (17.0)	4.375	0.112
Yes	153 (51.2)	85 (28.4)	61 (20.4)		
Primary cause, *n* (*%*)				1.836	0.986
Glomerulonephritis	42 (51.9)	23 (28.4)	16 (19.8)		
Diabetic nephropathy	37 (46.8)	24 (30.4)	18 (22.8)		
Hypertensive nephropathy	43 (51.8)	23 (27.7)	17 (20.5)		
Polycystic kidney/nephrotic syndrome	15 (53.6)	7 (25.0)	6 (21.4)		
unknown	17 (50.0)	12 (35.3)	5 (14.7)		
Duration of MHD since diagnosis, years, *n* (*%*)				2.758	0.839
< 2	32 (50.8)	17 (27.0)	14 (22.2)		
2–5	75 (49.7)	45 (29.8)	31 (20.5)		
5–10	25 (47.2)	19 (35.8)	9 (17.0)		
> 10	22 (57.9)	8 (21.1)	8 (21.1)		
Serum phosphorus (mmol/L), *M* (*P*_25_, *P*_75_)	1.83 (1.49, 2.22)	1.70 (1.38, 2.24)	1.56 (1.18, 2.04)	7.345	0.025
Serum calcium (mmol/L), *M* (*P*_25_, *P*_75_)	2.27 (2.19, 2.42)	2.30 (2.12, 2.44)	2.27 (2.12, 2.40)	0.347	0.841
Hemoglobin (g/L), *M* (*P*_25_, *P*_75_)	112.50 (102.0, 121.25)	109.0 (97.0, 119.0)	103.0 (91.0,116.5)	7.962	0.019
Intact parathyroid hormone (pg/ml), *M* (*P*_25_, *P*_75_)	157.30 (86.05, 279.03)	181.70 (79.76, 311.05)	173.0 (110.13, 284.43)	1.192	0.551
Clearance index of urea (Kt/V), *M* (*P*_25_, *P*_75_)	1.45 (1.32, 1.64)	1.47 (1.25, 1.67)	1.49 (1.25, 1.64)	0.007	0.996
Overall symptom burden, *Mean* ± *SD*	12.27 ± 4.55	15.73 ± 4.11	20.45 ± 3.07	87.558	< 0.001
Overall symptom severity, *Mean* ± *SD*	23.19 ± 9.10	32.88 ± 10.17	49.19 ± 10.0	163.662	< 0.001
KPS, *M* (*P*_25_, *P*_75_)	90.0 (90.0, 90.0)	80.0 (60.0, 90.0)	60.0 (50.0, 70.0)	118.103	< 0.001
PHQ-9, *M* (*P*_25_, *P*_75_)	4.0 (2.0, 6.0)	7.0 (5.0, 9.0)	13.0 (10.0, 15.0)	157.574	< 0.001
SSRS, *M* (*P*_25_, *P*_75_)	35.0 (29.8, 42.0)	31.0 (27.0, 36.0)	26.0 (24.0, 28.0)	81.162	< 0.001

Class 1 = mild palliative care needs, Class 2 = moderate palliative care needs, Class 3 = severe palliative care needs, *POS* Palliative care outcome scale, *MHD* Maintenance hemodialysis, *KPS* Karnofsky performance status scale, *PHQ-9* Patient health questionnaire-9 item, *SSRS* Social support rate scale

**Table 5:** Multinomial logistic regression analysis of the POS among three classes (*N* = 305)

**Variables**	**Class 1 vs. Class 3** ^a^				**Class 2 vs. Class 3** ^a^				**Class 2 vs. Class 1** ^a^			
	***β***	***SE***	***P***	***OR***	***β***	***SE***	***P***	***OR***	***β***	***SE***	***P***	***OR***
Intercept	-3.884	4.457	0.384	-	-2.303	4.128	0.577	-	1.581	2.090	0.449	-
Age, years												
< 65	-0.462	0.861	0.592	0.630	-0.671	0.782	0.391	0.511	-0.210	0.412	0.611	0.811
≥ 65 ^a^												
Education level												
Junior high school and below	-1.356	1.033	0.189	0.258	-1.423	0.934	0.128	0.241	-0.067	0.476	0.888	0.935
Senior high school	-3.387	1.345	0.012	0.034	-3.425	1.274	0.007	0.033	-0.038	0.496	0.938	0.962
Senior high school above ^a^												
Place of residence												
Rural	0.784	1.445	0.587	2.191	2.094	1.181	0.076	8.117	1.310	0.853	0.125	3.705
Urban ^a^												
Per capita monthly household income												
< 2,000 Yuan RMB (approximately 280.4341 US$)	-5.899	1.310	< 0.001	0.003	-3.101	1.213	0.011	0.045	2.798	0.553	< 0.001	16.410
2,000–5,000 Yuan RMB (approximately 280.4341–701.0853 US$)	-0.149	1.091	0.891	0.862	0.546	1.035	0.598	1.726	0.695	0.434	0.109	2.003
> 5,000 Yuan RMB (approximately 701.0853 US$) ^a^												
Serum phosphorus (mmol/L)	-0.093	0.402	0.817	0.911	-0.073	0.349	0.834	0.929	0.020	0.235	0.932	1.020
Hemoglobin (g/L)	0.038	0.024	0.118	1.039	0.030	0.022	0.169	1.031	-0.007	0.011	0.515	0.993
Overall symptom burden	-0.057	0.152	0.707	0.944	-0.153	0.139	0.272	0.858	-0.096	0.075	0.205	0.909
Overall symptom severity	-0.202	0.060	0.001	0.817	-0.087	0.050	0.085	0.917	0.116	0.037	0.002	1.123
KPS	0.140	0.031	< 0.001	1.151	0.092	0.028	0.001	1.097	-0.048	0.016	0.002	0.953
PHQ-9	-0.593	0.166	< 0.001	0.553	-0.505	0.150	0.001	0.603	0.088	0.080	0.273	1.092
SSRS	0.301	0.097	0.002	1.351	0.280	0.093	0.003	1.323	-0.021	0.029	0.461	0.979

^a^ Reference group, Class 1 = mild palliative care needs, Class 2 = moderate palliative care needs, Class 3 = severe palliative care needs; POS palliative care outcome scale, KPS karnofsky performance status scale, PHQ-9 patient health questionnaire-9 item, SSRS social support rate scale

## Discussion

### Classification characteristics of palliative care needs in MHD patients

Previous studies mostly judged the level of palliative care needs based on the total POS score, without considering the heterogeneity of the population [20, 27, 29]. To our knowledge, it might be the first latent profile analysis of the POS. In this study, the palliative care needs of MHD patients were divided into three categories, including mild, moderate and severe palliative care needs, and the proportions of each group were 50.5*%*, 29.2*%* and 20.3*%*, respectively, which means that nearly half of the patients had moderate and severe palliative care needs. Furthermore, this study found that family anxiety was more prominent among unmet needs, which was consistent with previous findings [14]. In particular, it is important to emphasize that the practical need scores of the moderate-to-severe palliative care need group were relatively high in our study (Additional file: Table S1). In a word, classifying the level of palliative care needs can not only help to identify priority care groups, but also clarify the status of various needs within groups, so that they can be provided with personalized care as early as possible.

### Influencing factors of unmet needs in MHD patients

Previous studies on the factors of patients' unmet needs have mostly focused on the individual level [20, 44]. After potential profile analysis, based on the socio-ecological model, we noted that MHD patients' unmet needs were mainly affected by education level, financial stress, functional status, symptom burden, and social support.

### Education level

This study found that patients with lower levels of education had more unmet needs, it was consistent with previous findings in patients with advanced cancer [45]. The consideration reason may be that patients with low education levels are more likely to have poor treatment adherence and health outcomes, resulting in their higher needs. Specifically, in order to prevent the occurrence of adverse health outcomes such as hospitalization rate and mortality, MHD patients must have good treatment compliance [46]. Treatment compliance is mainly reflected in medication, diet and lifestyle, which meets the requirements of health care providers [47]. As is known to all, adequate health literacy is the premise of high adherence [48]. However, MHD patients often experience complex treatment-related information, resulting in limited health literacy, which is especially obvious in patients with low education level [49, 50]. This suggested that the individual situation of the patient should be considered throughout the disease trajectory, proactively assessing their unmet needs and providing specialized support, which inevitably requires the involvement of a multidisciplinary team. Therefore, it is particularly urgent to establish an interdisciplinary team composed of nephrologists, nurses, dietitians, etc., to provide holistic care for patients [51].

### Financial stress

Although our respondents were from more economically developed areas in China and their health insurance status was better, the results still showed that the unmet needs were greater among patients with low family income. Financial considerations include in-hospital costs such as dialysis, medication, tests and hospitalisation, as well as out-of-hospital costs such as nutrition, care, transport and accommodation. These direct and indirect costs impose a heavy financial burden on patients and their families [52]. Some previous studies noted that palliative care services play an important role in reducing the length of hospital stay and the high costs associated with unplanned hospitalizations and intensive medical treatments [11, 53]. Thus, at the policy level, in addition to encouraging the medical insurance department and relevant government departments to increase financial input and management, clinical practice guidelines for kidney palliative care in China should also be formulated to help medical staff identify the best treatment and referral time, in order to reduce the waste of medical resources and patients' economic burden.

### Functional status

The results of this study indicated that decreased functional status tend to increase the needs of MHD patients, it was consistent with previous findings in patients with amyotrophic lateral sclerosis [54]. Decreased functional status was an important signal of shortened survival, which can early discover the risk of death in ESRD patients [55]. A previous study investigated the changes in the disease trajectory of ESRD patients in the last year of life and found that the POS score of ESRD patients in the last month of life was twice that of the baseline [56], which proved that the unmet needs of patients increased significantly with the shortening of survival time. Accordingly, it is of necessity to regularly assess the functional status of patients to identify high-risk groups at an early stage, and actively communicate with patients and their families to help them choose appropriate treatment modalities, so as to maximize the treatment benefits, especially to help patients realize the desire for peace at the end of life [57].

### Symptom burden

This study found that patients with higher severity of symptoms tend to have greater needs, which was similar to findings in patients with cystic fibrosis [58]. The dual effects of the disease itself and the treatment led to a variety of symptoms in MHD patients. The average symptom in this study was 14.94 (SD = 5.22), which was similar to the results of Fleishman et al. [34]. Common symptoms included dry mouth, fatigue and pruritus (The prevalence and severity of each symptom were shown in Table S2). Symptom management is the main domain of kidney palliative care [59]. An prospective observational cohort study showed that the symptom burden of dialysis patients receiving palliative care improved compared to those receiving usual care [10]. In addition to various physical symptoms, psychological symptoms such as depression were also found to be another important factor affecting patients' unmet needs in this study. A previous study included depression in the symptom cluster of MHD patients, and pointed out that depression, like the symptoms of other body systems, brought high intensity pain to patients [60]. Therefore, medical staff should actively assess the physical and psychological symptoms of patients, and implement symptom management according to the assessment results. According to the recommendations of the National Kidney Foundation in Kidney Supportive Care: Core Curriculum 2020, approaches to symptom management include assessing causes, reversible factors, the degree of distress caused by symptoms, non-pharmacological and pharmacological intervention options, expectation management, and recognition of limitations of treatment [51].

### Social support

Social support theory points out that social support network can play an important buffering role in the face of stressors and enhance the ability of individuals to resist risks [61]. The results of this research revealed that patients with low levels of social support had greater unmet needs. Considering the reason may be that, on the one hand, family members not only provide daily care for patients, but also provide emotional support, so patients who lack family support strengthen the need for care. On the other hand, long-term HD treatment limits patients' social activities, leading to a large part of patients' lives being occupied by the disease, and also exacerbates patients' needs, which was consistent with the results of previous interviews with MHD patients' experiences [4]. In summary, family members should be encouraged to participate in the whole process of disease treatment of patients and face the disease challenges together with patients. It is also worth noting that patients should be inspired to return to society without conflict between disease and treatment, and take the initiative to participate in social activities, so as to improve their enthusiasm for life.

### Limitations

This study has the following limitations. First of all, the participants of this study were ESRD patients treated with HD, while the situation of patients choosing other treatment modalities is not known, and survey respondents were selected by convenience sampling at two HD centers, which may lead to sampling bias. Secondly, although it has been stated in the POS application instructions that this tool can be used to investigate the palliative care needs of patients with a chronic and progressive disorder [28], and was verified to have good reliability and validity [56, 62]. However, this tool is still not a special scale for patients with kidney disease, the palliative care needs tool specific for kidney disease could be used for investigation in the future. Thirdly, although this study included variables based on the social ecological model, there may still be relevant variables that were not included, and qualitative research methods can be used to further explore the factors of unmet palliative care needs of patients. Fourthly, there are many common segmentation strategies, such as cluster analysis, latent class analysis, latent class growth analysis, etc. Other segmentation strategies should be used in the future to compare with the results of this study [63]. Finally, the cross-sectional study design used in this study cannot reflect the causal relationship of the conclusions, and further longitudinal studies are needed to examine the direction of this relationship.

## Conclusions

In conclusion, nearly half of ESRD patients undergoing MHD had moderate to severe palliative care needs in China, and the unmet needs were mainly influenced by education level, financial stress, functional status, symptom burden, and social support. In the future, it is necessary to identify the priority populations for palliative care in clinical practice, as well as provide holistic care to them through interdisciplinary teams to meet their needs.

### Supplementary Information

Below is the link to the electronic supplementary material.

**Additional file 1:** **Table S1.** Comparison of POS scores among three groups (*N* = 305). **Table S2.** Prevalence and severity of individual symptoms (N = 305). **Table S3.** Multiple linear regression analysis of the POS (N = 305).

## Data Availability

The datasets used and analyzed during the current study are available from the corresponding author on reasonable request.

## Abbreviations

HD: Hemodialysis
MHD: Maintenance hemodialysis
ESRD: End-stage renal disease
HRQOL: Health-related quality of life
eGFR: Estimated glomerular filtration rate
POS: Palliative care outcome scale
DSI: Dialysis symptom index
KPS: Karnofsky performance status scale
PHQ-9: Patient health questionnaire-9 item
DSM-IV: Diagnostic and statistical manual of mental disorders
SSRS: Social support rate scale
LPA: Latent profile analysis
AIC: Akaike information criterion
BIC: Bayesian information criterion
SABIC: Sample-size-adjusted bayesian information criterion
LMR: Lo-mendell-rubin
BLRT: Bootstrap likelihood ratio test
ANOVA: One-way analysis of variance
