# Supplementary material for: Exploring the influencing factors of unmet palliative care needs in Chinese patients with end-stage renal disease undergoing maintenance hemodialysis: a cross-sectional study
Source: BMC Palliat Care. 2023 Aug 5;22:113. doi: 10.1186/s12904-023-01237-x (PMC10403855; doi:10.1186/s12904-023-01237-x)
Supplement: Supplementary file 1 — Additional file 1: Table S1. Comparison of POS scores among three groups (N = 305). Table S2. Prevalence and severity of individual symptoms (N = 305). Table S3. Multiple linear regression analysis of the POS (N = 305). [file 12904_2023_1237_MOESM1_ESM.pdf]

Additional file

Table S1 Comparison of POS scores among three groups (N = 305)

Item	Class 1 (n = 154)	Class 2 (n = 89)	Class 3 (n = 62)	H	P
Total	12.0（9.0, 15.0）	19.0（17.5, 22.5）	29.0（26.0, 31.0）	222.746	＜0.001
Pain	1.0（0.0, 1.0）	1.0（1.0, 2.0）	2.0（1.0, 3.0）	74.994	＜0.001
Other symptoms	1.0（1.0, 2.0）	2.0（1.0, 2.0）	3.0（2.0, 3.0）	113.789	＜0.001
Patient anxiety	1.0（0.0, 1.0）	1.0（1.0, 2.0）	2.0（2.0, 3.0）	145.420	＜0.001
Family anxiety	2.0（1.0, 4.0）	4.0（2.0, 4.0）	4.0（4.0, 4.0）	39.051	＜0.001
Information needs	2.0（1.0, 2.0）	2.0（1.0, 3.0）	4.0（2.0, 4.0）	43.861	＜0.001
Support	1.0（1.0, 3.0）	2.0（1.0, 3.0）	3.0（3.0, 4.0）	68.849	＜0.001
Depression	0.0（0.0, 1.0）	1.0（1.0, 2.0）	3.0（2.0, 3.0）	165.914	＜0.001
Self-worth	1.0（1.0, 1.0）	1.0（1.0, 2.0）	3.0（2.0, 3.0）	159.046	＜0.001
Wasted time	0.0（0.0, 2.0）	2.0（0.0, 2.0）	2.0（0.0, 4.0）	13.827	0.001
practice matters	0.0（0.0, 0.0）	4.0（3.0, 4.0）	4.0（4.0, 4.0）	256.850	＜0.001

Note: Data are M (P_{25}, P_{75}); POS palliative care outcome scale.

Table S2 Prevalence and severity of individual symptoms (N = 305)

Symptom	Prevalence<br>*n (%)* [a]	Severity<br>M (P_{25}, P_{75})[b]
Dry mouth	91.8	2.0（2.0, 3.0）
Feeling tired or lack of energy	85.2	2.0（1.0, 3.0）
Itching	85.2	2.0（1.0, 3.0）
Dry skin	84.9	2.0（1.0, 3.0）
Trouble staying asleep	75.1	2.0（0.5, 3.0）
Worrying	71.1	1.0（0.0, 2.0）
Trouble falling asleep	68.2	2.0（0.0, 3.0）
Feeling anxious	65.9	1.0（0.0, 2.0）
Feeling irritable	58.7	1.0（0.0, 2.0）
Decreased interest in sex	57.4	2.0（0.0, 2.0）
Difficulty becoming sexually aroused	56.7	2.0（0.0, 3.0）
Muscle cramps	55.7	1.0（0.0, 2.0）
Feeling sad	55.4	1.0（0.0, 2.0）
Bone or joint pain	53.4	1.0（0.0, 2.0）
Lightheadedness or dizziness	46.9	0.0（0.0, 2.0）
Feeling nervous	46.2	0.0（0.0, 2.0）
Muscle soreness	45.6	0.0（0.0, 2.0）
Difficulty concentrating	45.6	0.0（0.0, 2.0）
Constipation	44.6	0.0（0.0, 2.0）
Decreased appetite	41.3	0.0（0.0, 2.0）
Cough	39.7	0.0（0.0, 1.0）
Shortness of breath	39.3	0.0（0.0, 2.0）
Headache	35.1	0.0（0.0, 1.5）
Numbness or tingling in feet	29.5	0.0（0.0, 1.0）
Nausea	26.6	0.0（0.0, 1.0）
Chest pain	20.7	0.0（0.0, 0.0）
Vomiting	18.0	0.0（0.0, 0.0）
Swelling in legs	16.4	0.0（0.0, 0.0）
Restless legs or difficulty keeping legs still	16.4	0.0（0.0, 0.0）
Diarrhea	15.1	0.0（0.0, 0.0）

Note: [a] Percentage of patients who reported the symptom; [b] Based on the five-point Likert scale, 1 = “not at all bothersome” to 5 = “bothers very much”.

Table S3 Multiple linear regression analysis of the POS (N = 305)

Variables	B	SE	β	*t*	*P*	95% *CI*
Constant	26.625	2.861	-	9.306	＜0.001	（20.994, 32.255）
Per capita monthly household income	-1.232	0.281	-0.132	-4.388	＜0.001	（-1.784, -0.679）
Hemoglobin	-0.023	0.011	-0.053	-2.060	0.040	（-0.046, -0.001）
Overall symptom severity	0.223	0.024	0.409	9.342	＜0.001	（0.176, 0.270）
KPS	-0.105	0.015	-0.226	-6.781	＜0.001	（-0.135, -0.074）
PHQ-9	0.316	0.078	0.191	4.039	＜0.001	（0.162, 0.470）
SSRS	-0.156	0.031	-0.156	-5.019	＜0.001	（-0.046, -0.001）

Note: POS palliative care outcome scale, KPS karnofsky performance status scale, PHQ-9 patient health questionnaire-9 item, SSRS social support rate scale.
